# Multi-Scale-Porosity TiO_2_ scaffolds grown by innovative sputtering methods for high throughput hybrid photovoltaics

**DOI:** 10.1038/srep39509

**Published:** 2016-12-21

**Authors:** Salvatore Sanzaro, Emanuele Smecca, Giovanni Mannino, Corrado Bongiorno, Giovanna Pellegrino, Fortunato Neri, Graziella Malandrino, Maria Rita Catalano, Guglielmo Guido Condorelli, Rosabianca Iacobellis, Luisa De Marco, Corrado Spinella, Antonino La Magna, Alessandra Alberti

**Affiliations:** 1 National Research Council-Institute for Microelectronics and Microsystems (CNR-IMM), Zona Industriale-Strada VIII no. 5, Catania 95121, Italy; 2 University of Messina, Department of Mathematical and Computational Sciences, Physics and Earth Sciences, V. le F. Stagno d’Alcontres 31, Messina 98166, Italy; 3 Department of Chemical Sciences, University of Catania, V. le Andrea Doria 6, 95125 Catania, Italy; 4 Italian Institute of Technology Foundation-Center for Biomolecular Nanotechnology (IIT-CBN) Via Barsanti sn, 73010, Arnesano, Italy; 5 University of Salento, Department of Innovation Engineering, Via per Monteroni 73100, Lecce, Italy; 6 National Research Council-Institute of Nanotechnology (CNR-Nanotec), District of Technology, Via Arnesano 16, 73100 Lecce, Italy

## Abstract

We propose an up-scalable, reliable, contamination-free, rod-like TiO_2_ material grown by a new method based on sputtering deposition concepts which offers a multi-scale porosity, namely: an intra-rods nano-porosity (1–5 nm) arising from the Thornton’s conditions and an extra-rods meso-porosity (10–50 nm) originating from the spatial separation of the Titanium and Oxygen sources combined with a grazing Ti flux. The procedure is simple, since it does not require any template layer to trigger the nano-structuring, and versatile, since porosity and layer thickness can be easily tuned; it is empowered by the lack of contaminations/solvents and by the structural stability of the material (at least) up to 500 °C. Our material gains porosity, stability and infiltration capability superior if compared to conventionally sputtered TiO_2_ layers. Its competition level with chemically synthesized reference counterparts is doubly demonstrated: in Dye Sensitized Solar Cells, by the infiltration and chemisorption of N-719 dye (∼1 × 10^20^ molecules/cm^3^); and in Perovskite Solar Cells, by the capillary infiltration of solution processed CH_3_NH_3_PbI_3_ which allowed reaching efficiency of 11.7%. Based on the demonstrated attitude of the material to be functionalized, its surface activity could be differently tailored on other molecules or gas species or liquids to enlarge the range of application in different fields.

A bright future of our society is based on the capability of tuning the material properties for beneficial scopes. TiO_2_–based 1D nanostructures are especially appealing to combine the advantages of exposing large active facets^1^,^2^ with good mechanical performances and high electrical conductivity^3^. All those properties enrich 1D-TiO_2_ architectures with a transversal empowering for energy, health, and environment applications, since they can be efficiently used as scaffold for Dye^4^ and Perovskites Solar Cells^5^, anodes for Li batteries^6^, electrodes for water splitting devices^7^, materials for biosensors detecting DNA^8^ or glucose^3^, scaffold for gas sensing^9^,^10^, and scaffold for photo-catalysis^11^,^12^.

Among the possible applications that can take advantage from the use of TiO_2_ nanostructures having multi-scale-porosity, DSCs (Dye Solar Cells)^13^,^14^ have been pushed to a high level of maturity and, nowadays, represent an interesting alternative to semiconductors technologies mainly due to the large versatility and the low production cost^15^,^16^. Using DSC technologies, the light-to-current conversion efficiency was raised up to 13%^17^. More recently, hybrid solar cells replacing the dye with hybrid Perovskites (PSC) materials^18^,^19^,^20^,^21^,^22^ as sensitizer allowed getting efficiency values as high as 22.1% using advanced mixed perovskites^23^,^24^. In the standard scheme of the photo-anode of a DSC^4^ or PSC^18^, a mesoporous thin film of nanosized TiO_2_ crystals is deposited on a Transparent Conductive Oxide (TCO), annealed for grains sintering and anatase crystallization (typically at 500 °C), and subsequently imbued with a photoactive dye^25^,^26^,^27^,^28^,^29^ or infiltrated with perovskite^30^. In both cases, mesoporous TiO_2_ in the anatase polymorphism has emerged as the most appropriate choice for cell scaffold^15^,^21^,^23^,^31^. The most diffused and versatile way to generate nano-TiO_2_ architectures is by chemical approaches. They offer a large plethora of fascinating hierarchical and mesoporous structures^32^,^33^,^34^ with high infiltration capability.

On the other hand, standard physical growth methods cannot straightforwardly compete with chemical approaches since they have an intrinsic tendency to form compact layers. The advent of modified sputtering methodologies to grow, instead, TiO_2_ scaffolds with high porosity levels would open the field to high production throughput as linked to the reproducibility of the materials and to the up-scalability of the processes. A first attempt was provided by Thornton with his semi-empirical model^35^,^36^. The Thornton’s model defines a range of deposition parameters such that the layer structure can be qualitatively tuned from dense to nano-porous. On the basis of this model, the deposition temperature must be taken as low as possible to reduce surface diffusion of the deposited ad-atoms, and the Ar pressure likely high enough to increase the layer porosity. Nonetheless, the pore size is extremely limited. Several attempts were done in the literature in order to increase the TiO_2_ layer porosity above the Thornton limit by using sputtering or evaporation approaches which generated cauliflower^37^, penniform^38^ or zig-zag^39^ structures. Some other attempts were done by GLancing Angle Deposition (GLAD)^40^ to deposit Ti nanostructures^41^,^42^,^43^ which requires *ex-situ* oxidation for TiO_2_ reaction; or by using array of template materials (e.g. polystyrene nano-spheres) to exploit their shadowing effect during TiO_2_ growth^44^. They however lack of process simplification and of a transversal demonstration on the applicative valence of the porous scaffold to largely and deeply infiltrate materials of different size and/or nature to be used for wide-ranging applications. This evidence must be also corroborated by the demonstration of an effective interconnection between the host (scaffold) and the guest (infiltrated materials) to exchange information (e.g. carriers).

In this paper we propose a material with multi-scale porosity ranging from nano- to meso- dimensionalities grown by a modified (up-scalable) sputtering method, and we demonstrate its attitude to be functionalized with species of different nature. To this intent, two photoactive blends, namely TiO_2_+ dye and TiO_2_+ perovskite, are realised, characterised and tested in prototype DSCs and PSCs to provide a proofs-of-concept on the transversal empowering of the material.

## Results and Discussion

### Grazing incidence-local oxidation (gig-lox) process description

**Figure 1:**
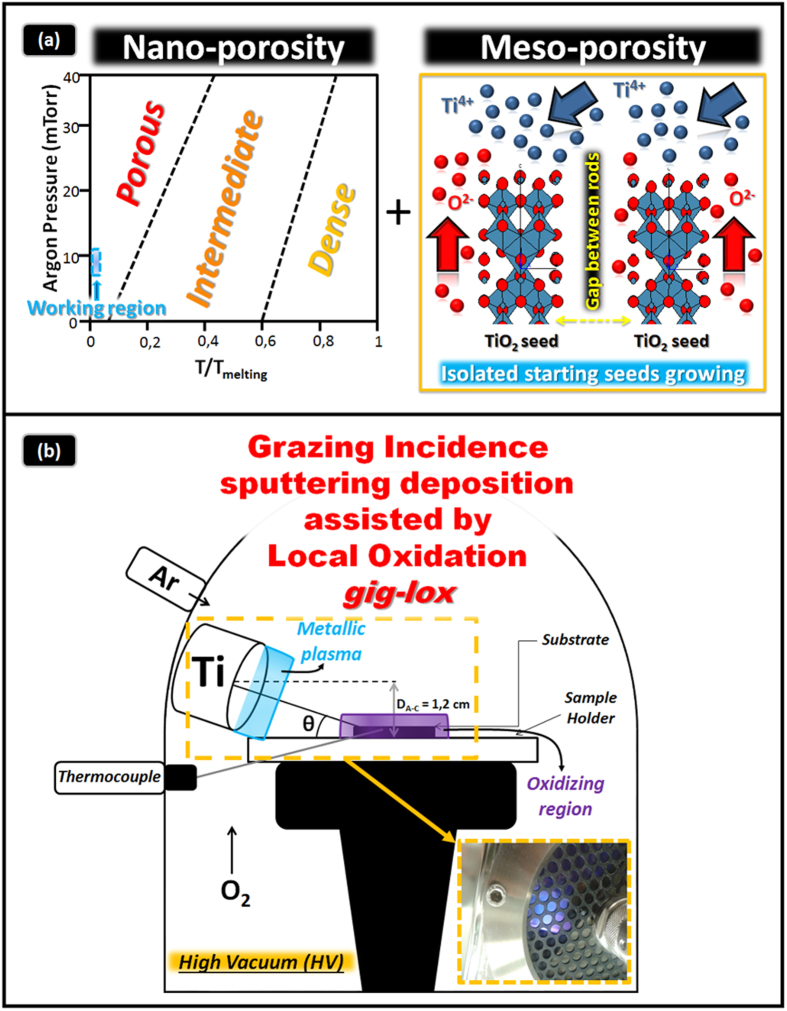
Schematic representing the combined methodology to deposit TiO_2_ materials with a multi-scale porosity. (**a**) Left panel: nano-porosity expected by the Thornton’s model; right panel; meso-porosity achieved by a combination of shadowing effects plus local oxidation. The seeds are formed at the early stages of the deposition process. (**b**) Customized Sputtering system which allows working in a separated-charged-species regime combined with shadowing effects. θ is the inclination angle of the source. The inset is a picture of the double plasma (cyan = metallic and violet = oxidizing) established inside the chamber.

The idea to build the new material is sketched in Fig. 1(a). It is based on the exploitation of a double strategy to provide the material with a double-range-porosity during growth (*in-situ* processes).

A nano-porosity (1–5 nm) was settled by applying Thornton’s conditions^35^. Our specific working area is indicated in Fig. 1(a) (left), with the argon pressure raised up to 10.5 mTorr, and the deposition done at room temperature to reduce diffusive effects. To reach a higher porosity (>5 nm) we used a customized sputtering equipment empowered by a Ti off-axis source with an inclination angle θ (Fig. 1(b)) and an oxidizing zone confined at the sample surface, far from the cathode sheath region.

The success in growing a layer with meso-porosity extending through thick layers (e.g. through 1000 nm) mainly resides in the choice of the inclination angle θ (see Supplementary Information Fig. 1S). An optimum inclination angle of 12.7° was identified in our setup as a compromise between the shadowing effect by the starting TiO_2_ seeds (this define the lower limit in θ) and the verticalization of the Ti flux by increasing θ, that has a tendency to progressively close the pores by proceeding the deposition. Hereafter we refer to our refined methodology at inclination angle of 12.7° as gig-lox; the reference process, related to the use of a standard parallel plate geometry in Thornton conditions^45^, will be indicated by ppg. See methods and SI for more details.

### Morphological analyses

**Figure 2:**
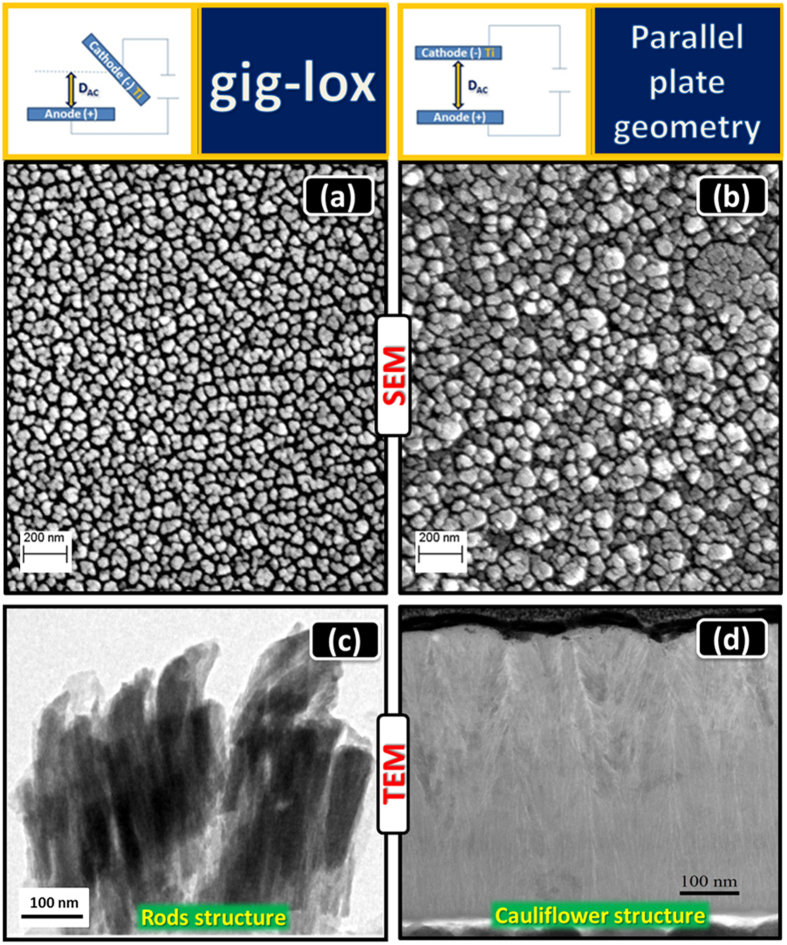
Morphological analyses of the as prepared samples. FE-SEM images in plan-view of TiO_2_ (**a**) in gig-lox and (**b**) in ppg configurations. Cross section TEM images of TiO_2_ (**c**) gig-lox rods and (**d**) ppg grains having a cauliflower structure. In the gig-lox layer the grains size distribution is more uniform than in the ppg material.

The morphology of the Ti-oxide layers was evaluated by Field Emission-Scanning Electron Microscopy (FE-SEM) in plan-view and cross section configurations (Fig. 2(a,b)). To avoid charging effects, Au nanograins (∼5 nm in diameter) were sputtered on the sample surface or section (in some cases they are visible on the sample surface). The as deposited gig-lox layer (Fig. 2(a))is characterized by an array of grains, whose diameter is in the range of ∼60–90 nm (Supplementary Information Fig. 2S). For the starting material composition see XPS results in the Supplementary Information, Fig. 3S.

The TiO_2_ grains are surrounded by a network of voids. As counterpart, the as deposited ppg layer, shown in Fig. 2(b), is made of grains with larger size (∼50–150 nm) often aggregated in form of cauliflowers. The TEM cross sections of the two layers are compared in Fig. 2(c,d). The gig-lox layer is made of separated rods extending through the whole thickness. On the other hand, the ppg layer is more compact especially at the bottom half part. The uppermost part has instead a typical cauliflower morphology often reported in the literature^37^, which produces a high surface roughness (±20 nm) in correspondence of the boundaries between adjacent grains.

**Figure 3:**
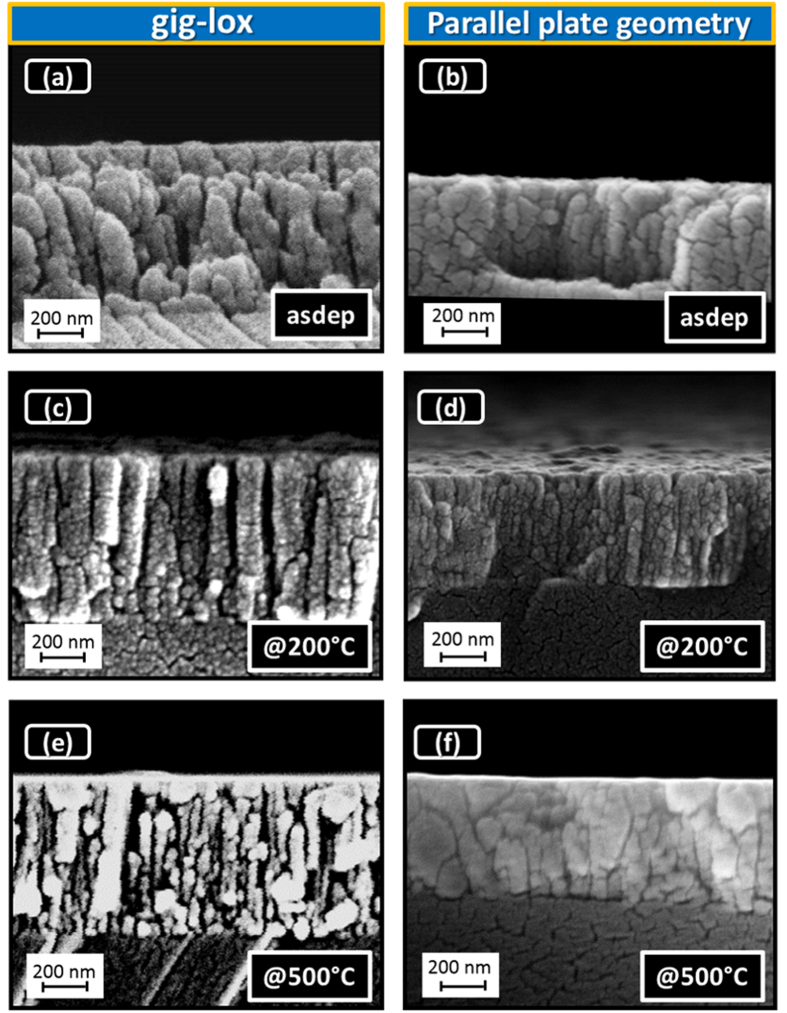
Cross-section FE-SEM images of TiO_2_ layers deposited. TiO_2_ (**a**) in gig-lox (∼800 nm) and (**b**) in ppg (∼500 nm) configurations. Layers after thermal treatment: at 200 °C 30 min in (**c**) gig-lox, (**d**) ppg layers; at 500 °C 30 s in (**e**) gig-lox, (**f**) ppg layers. The annealings were done in air. Notice how the gig-lox layer, differently from the reference, retains its deep porosity even after treatment at 500 °C.

Figure 3a visualizes a rods forest in the as deposited gig-lox layer, with rod diameter in the range of ∼80–90 nm, in agreement with their plan-view (Fig. 2(a)). The rods are separated by ∼10–50 nm large voids extending through the whole thickness (∼800 nm). This represents the meso-porosity of the layer. The ppg layer appears, instead, more compact, similarly to what reported in the literature^37^,^45^,^46^,^47^,^48^. Accordingly, after thermal treatments, grain coalescence causes a shrinkage of the pores in the ppg layer (especially at the surface: see Fig. 3(d,f)). This effect is more pronounced after annealing at 500 °C. On the other hand, the gig-lox layer retains its porosity through the whole thickness with the rod forest architecture still preserved.

### Porosity calculation from Ellipsometric measurements

**Figure 4:**
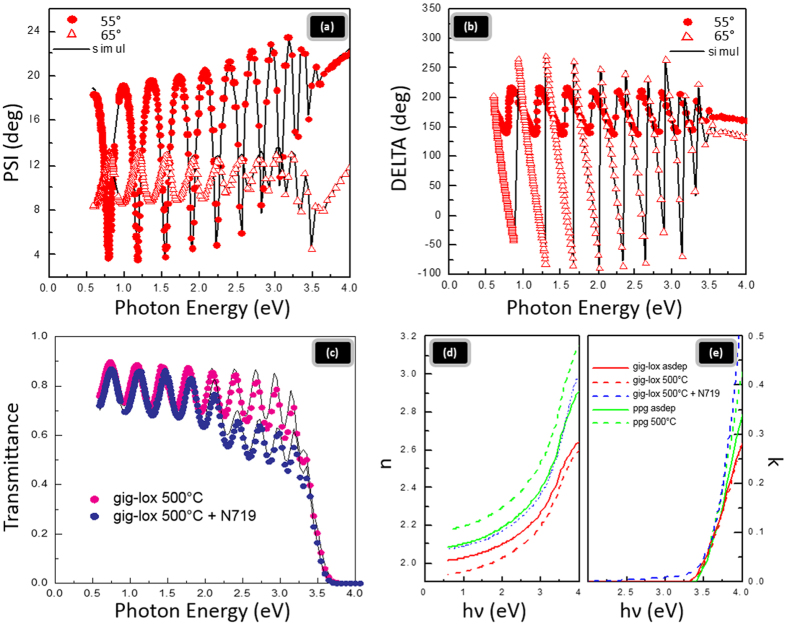
Ellipsometric measurements. Spectroscopic Ellipsometric measurements of experimental (symbols) and generated (lines) curves at 55° and 65° angles of incidence of Ψ **(a)**, and of Δ **(b)** for the gig-lox layer as deposited; (**c**) transmission data (symbols) and simulated (lines) for gig-lox layer annealed at 500 °C and gig-lox layer annealed at 500 °C + N-719, **(d,e)** the calculated refractive index (n) and extinction coefficient (k) of ppg and gig-lox layers before and after annealing at 500 °C and for the gig-lox layer after annealing at 500 °C + N-719.

The TiO_2_ porosity has been evaluated by SE measurements on ∼800 nm-thick samples. The values of n at 550 nm and the layer porosity (see methods for details) are available in Table 1. We found that as deposited ppg sample is less porous than the gig-lox one and it becomes much more compact when subjected to annealing at 500 °C. In contrast, the porosity in the gig-lox sample does not change with annealing because the nano-structure is preserved; its refractive index is rather reduced according to the structural arrangement of TiO_2_ in the anatase lattice^49^ (Fig. 4(d) and Table 1. See also XRD). As a related effect of the annealing, we measured an increase of the bandgap (see Fig. 4S) which has been associated to the improvement of the lattice quality. Regarding the transmittance, the gig-lox layer retains an average value of ∼80% in the visible range below the TiO_2_ bandgap (Fig. 4(c)).

### Structural analyses

**Figure 5:**
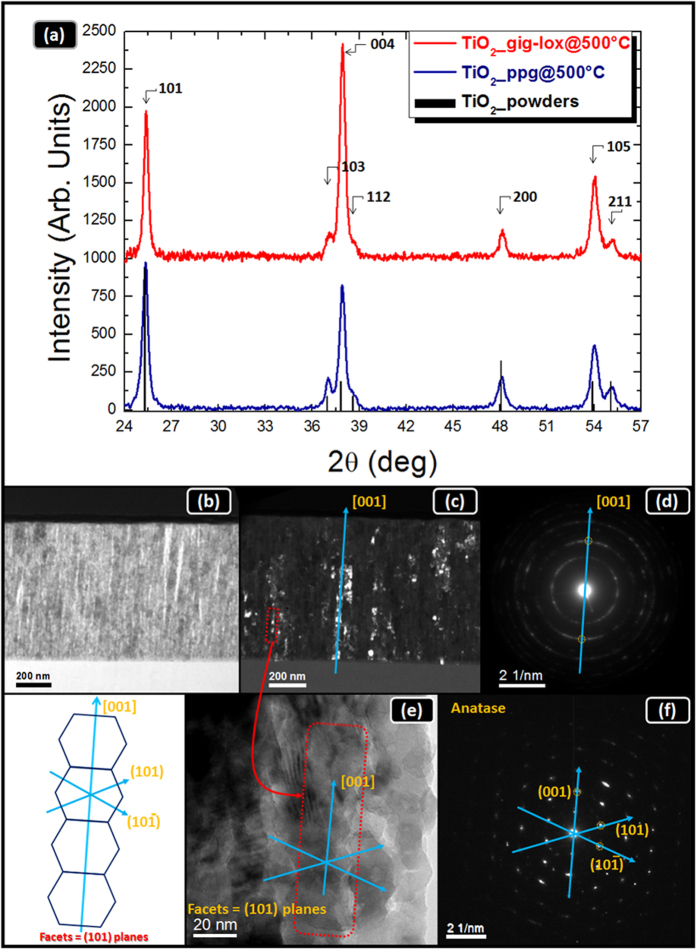
Structural analyses. (**a**) symmetric XRD patterns (2θ-ω) of TiO_2_ layers deposited in gig-lox (red) and in ppg (blue) configuration after thermal treatment at 500 °C. Both patterns refer to anatase. TEM analyses of the TiO_2_ gig-lox layer after annealing at 500 °C: (**b**) bright field image showing the meso-porous structure (the pores give a bright contrast); (**c**) dark field image originating from the region circled in (**d**), highlighting single grains stacked within the rods (the bright objects); (**e**) detail of a rod with the related SAED in (**f**). Left panel: a representative schematic of a TiO_2_ rod made of stacked grains with facets along (101) and (10-1) planes, with growth axis along the [001] direction, according to XRD data in (**a**). The preservation of this fine structure denotes that the annealing has a conservative effect on the porosity.

X-Ray diffraction analyses have confirmed that a structural improvement arises from the use of a thermal treatment. This improvement is progressively gained by raising the temperature from 200 °C to 500 °C (see Supplementary Information Fig. 5S). The final lattice structures of the gig-lox with respect to the ppg layer are compared in Fig. 5(a) after a thermal treatment at 500 °C^50^,^51^. We deduce a general predominance of the (004) growth planes in both materials. Despite this similarity, the gig-lox layer has a larger abundance of domains exposing (004) planes at the sample surface. The texturing values (J) calculated for all the detected crystallographic planes^31^ are reported in SI, Table 2S.

Most of the works in the literature on 1D-nano-structured TiO_2_ materials report the (101) as preferential growth planes^1^,^44^,^52^,^53^,^54^,^55^; in some other cases (220)^37^ or (004)^44^,^46^,^47^,^54^,^56^ planes were claimed as preferentially selected during the growth process. The different findings must be rationalized in the framework of the specific growth conditions/methods used, namely the anode-cathode distance, the oxygen concentration during deposition, the power loading, etc. The comparison of literature data elucidates that ad-atoms with high mobility are created under low pressure and/or high potential and/or high deposition temperature^57^, and this favours the exposure of (101) surfaces.

To go deeper into the growth process and to understand the origin of the preferential orientation of our gig-lox layer along the [001] direction, a complementary study on the structure was performed by Transmission Electron Microscopy. The meso-porosity of the layer is visible in Fig. 5(b) with the pores giving a bright contrast. The dark field image in Fig. 5(c), collected by selecting a small portion of the (004) planes (Fig. 5d), highlights a rod structure made of vertically stacked grains, with nano-porosity between them. The grains are laterally faceted as shown in Fig. 5e and f. Similar architectures made of stacked faceted grains were reported elsewhere^58^,^59^ for TiO_2_ synthesized by sol-gel procedures. In those cases, it was proposed that the self-assembling of nanostructures into branched rods is based on a surface free energy gain (thermodynamics) combined with kinetic factors. In particular, the self-assembling of TiO_2_ nanograins along the [001] growth axis is argued to originate from the inherent anisotropy (tetragonal unit cell) and the centro-symmetry (body-centered lattice) of the TiO_2_ anatase lattice. This promotes equi-directional linear development of the nanocrystal lattice mainly along the high symmetry c-axis. The concept can be properly applied to gig-lox and ppg growth processes. Specifically, in the ppg growth, the TiO_6_-based chain-like pattern can expand out of the main c-axis^58^ and generate the cauliflower structures frequently observed in thick layers^37^, likely due to the branching of the TiO_6_ blocks mainly along the equivalent transversal [101] and [011] directions^58^,^60^. In the gig-lox case instead, the shadowing prevents the TiO_6_ blocks to widely and freely expand in the lateral directions.

**Figure 6:**
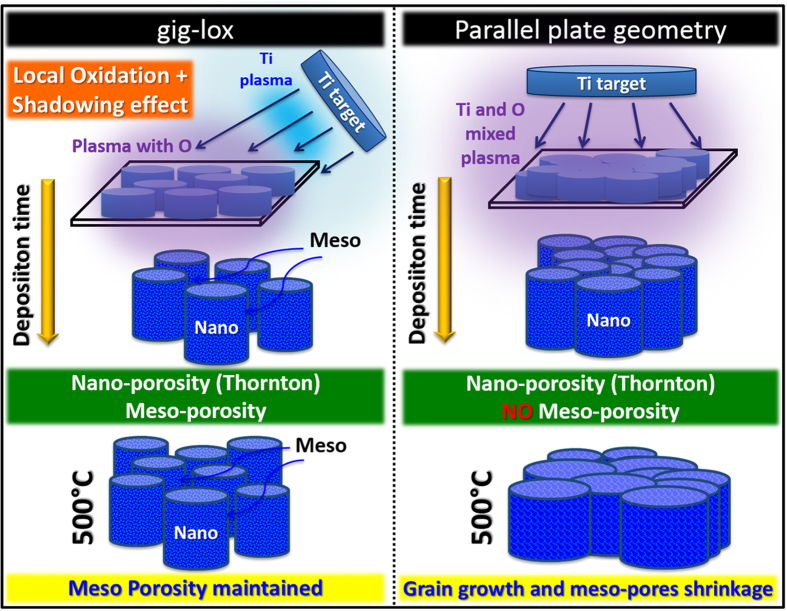
Representative schematic of gig-lox and ppg processes. The porosity of the gig-lox TiO_2_ layer is maintained even after annealing at 500 °C, while in the ppg TiO_2_ layer a dramatic pore shrinkage is observed after the same thermal treatment. Note also: (1) the double plasma regime used in gig-lox; (2) the double range porosity (nano and meso) achieved in gig-lox.

On the basis of what discussed, a general summarizing schematic is drawn in Fig. 6. In particular, it is evidenced the empowering introduced by the porosity retention in the gig-lox scheme after annealing with respect to the reference approach, due to the rods separation.

### Dye loading

**Figure 7:**
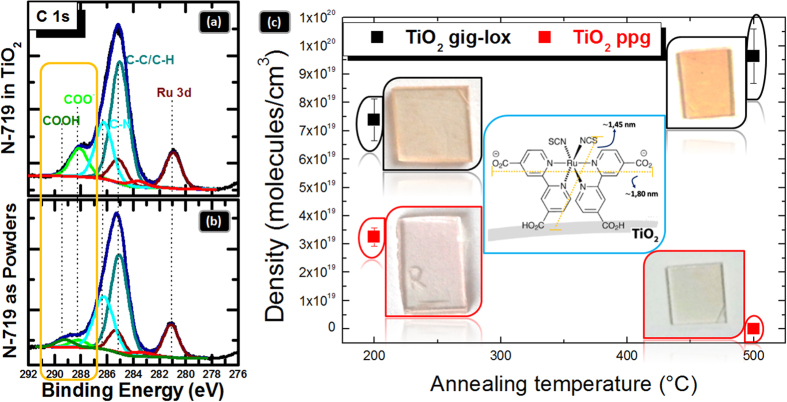
Analyses on the dye loading process. XPS high resolution spectra in the C 1 s region for (**a**) gig-lox TiO_2_ layer functionalized with N-719 compared to (**b**) N-719 powders. The comparison with the N-719 powders in (**b**) allows associating the contribution at 280.9 eV to the Ru 3d_5/2_ binding energy (the Ru 3d_3/2_ component is at 285.1 eV). The COO^−^ groups, giving contributions 288.2 eV, are used as markers for the molecular anchoring on the TiO_2_ surface; (**c**) density of molecules infiltrated into the differently pre-treated TiO_2_ layers (gig-lox vs. ppg). Insets: photos of functionalized TiO_2_ glasses with the colour depending on the molecular density; N-719 molecule structure and dimensions.

The empowering offered by the gig-lox structure was tested on its infiltration capability and surface reactivity by using test-molecules bearing a COOH group to be anchored on the TiO_2_ surfaces like N-719^4^, since largely used in the literature. X-Ray Photoelectron Spectroscopy was used to investigate the TiO_2_ surface modification after sensitization (Fig. 7(a)). The band due to the C-C/C-H species is located at 285.0 eV, whilst the C-N bond and the COO^−^ group give contributions at 286.3 eV and at 288.2 eV, respectively. As a fingerprint of the anchoring, the last component is more pronounced in the gig-lox than in the powders spectrum (Fig. 7(b)). As a further evidence of the molecular anchoring on the TiO_2_ surface, the band at 289.4 eV, due to the unreacted COOH groups, is not present in the spectrum of the infiltrated TiO_2_ layer. All those findings thus testify that dye chemisorption is occurred on the TiO_2_ surface^61^. Chemisorption is the pre-requisite for photo-electron injection from the dye to TiO_2_.

Spectroscopic Ellipsometry reveals the occurrence of some optical modifications after dye loading: an increase of the refractive index which was associated to the dye molecules filling the TiO_2_ pores (see Fig. 4(d) and Table 1); a reduction of the layer transmittance in the range of 2.2–3.5 eV, wherein the specific absorption on N-719 is expected (Fig. 4(c)); and, accordingly, an increase of the extinction coefficient in the tail below 3.5 eV (Fig. 4(e)).

By UV-Vis absorption measurements we estimate the density D_[N-719]_ using the Lambert-Beer law: 
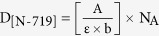
, where A is the absorbance, ε is the molar absorption coefficient, b is the light path and the N_A_ is the Avogadro’s number^62^. In our case, the molar absorption coefficient is ∼1.4 × 10^4^ M^−1^cm^−1 ^4 (see Table 3S). The data were also represented in Fig. 7(c) as a function of the annealing temperature done before sensitization. From the comparison it emerges that the gig-lox layer annealed at 500 °C accommodates the largest amount of molecules, likely due to the crystallographic improvement of the surface exposed by the TiO_2_ rods edges with respect to the starting material, comprising: a) the faceting of the external surfaces (see TEM in Fig. 5) and b) a likely increased reactivity of the anatase surfaces in comparison to less ordered surfaces^3^,^4^,^25^,^63^. Note that a value of ∼1 × 10^20^ molecules/cm^3^ is consistent with a complete pore filling as calculated by taking into account the full volume of the pores and the N-719 size (the volume occupied by each molecule was ∼3.8 nm^3^). On the other hand, a lower infiltration capability is offered by the ppg layer in the sample pre-treated at 200 °C due to the more compact overall structure (see Fig. 2d); moreover, grain coalescence definitively obstructs the dye infiltration in the sample pre-treated at 500 °C.

### Devices

**Figure 8:**
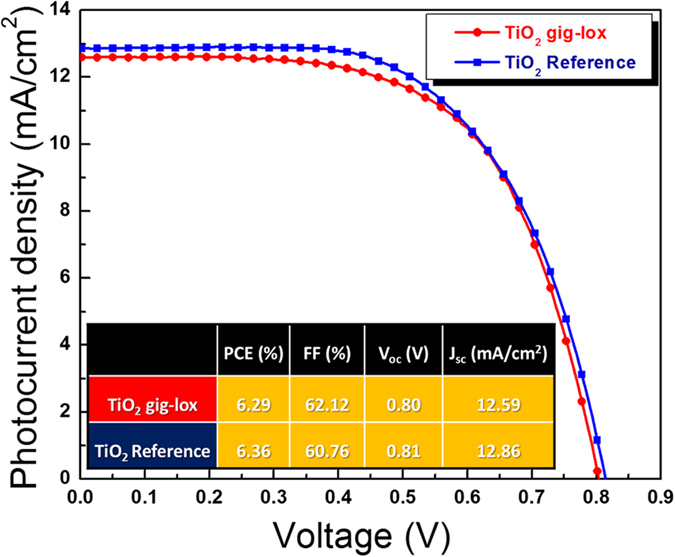
I–V curves of DSCs. Devices (∼1 μm-thick) based on gig-lox TiO_2_ layers compared to a reference made by commercial TiO_2_ pastes (Dyesol 18NR-T) under 1.0 sun illumination.

As a first proof of concept, we employed the described gig-lox material in the architecture of Dye Sensitized Solar Cells. The photocurrent-voltage characteristics are shown in Fig. 8.

They testify the successful combination of dye loading and chemisorption occurred into the TiO_2_ gig-lox scaffold. Photo-conversion efficiencies of 6.3% were measured for the gig-lox layers (see methods for details), showing performances comparable to the commercial reference device. Those values are positively considered if taken in relationship to the reduced thickness of the scaffold (we used 1 μm instead of ∼10 μm as commonly done) and to the low molar absorption coefficient of the N-719 dye^29^,^34^. This represents our first demonstration that a sputtered TiO_2_ layer aspires to compete with chemically prepared counterparts (integrated in the same architecture), additionally offering the up-scalability of the growth process as added-value.

**Figure 9:**
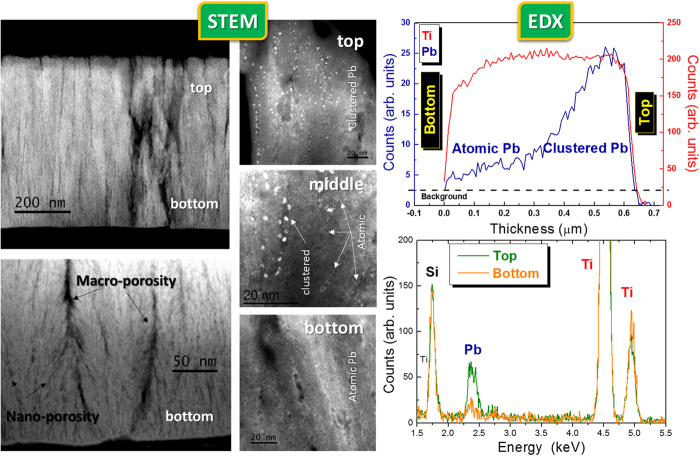
STEM and EDX analyses on the TiO_2_ gig-lox layer after CH_3_NH_3_PbI_3_ infiltration. In the STEM images, the Pb nano-aggregates (white Z-contrast) result from the perovskite degradation under high vacuum conditions^66^ and are used as markers of the original distribution of the perovskite layer. Notice how the Pb aggregates nicely stack into the pores; also follow the Pb atomic distribution into the bottom part of the layer. Here the Pb/Ti atomic ratio is ∼1/10, close to what expected by combining their atomic density in the starting CH_3_NH_3_PbI_3_ (∼3Pb/1nm^3^) and in the TiO_2_ layer with the gig-lox porosity (CH_3_NH_3_PbI_3_/TiO_2_ ∼40% in volume) in a regime of complete pore filling.

A second proof of concept was provided by integrating the TiO_2_ gig-lox layer as scaffold in Perovskite Solar Cells, using a solution processed CH_3_NH_3_PbI_3_ material. Consistently with what already shown, a 1 μm-thick TiO_2_ layer was investigated and the extent of infiltration by the perovskite evaluated^19^,^64^,^65^. In Fig. 9 we provide the demonstration of the successful deep interpenetration between our gig-lox scaffold and the solution processed CH_3_NH_3_PbI_3_. Since STEM is very sensitive to species with high atomic number (Z), it indeed gives evidence (mainly) of the spatial distribution of the Pb atoms. They are found to be atomically distributed through the layer or aggregated in nanometer spheres giving a bright contrast in the images. This results from (mainly) vacuum-induced degradation^66^,^67^ of the starting perovskite layer. Perovskite degradation occurs by creation and loss of volatile species^66^, and this causes iodine leaving the sample likely in form of HI. The consequent huge volume contraction^20^ with respect to the initial perovskite distribution is responsible for the spatial discontinuity of residual Pb species observed in Fig. 9. Nonetheless, the integral amount of Pb atoms, compared to the Ti counterpart (see EDX results in Fig. 9), accounts for a starting complete pore filling inside the TiO_2_ scaffold.

Although the information on the continuity of the perovskite coverage over the TiO_2_ rods is consequently lost, the distribution and location of the Pb agglomerates depict the capability of the perovskite to deeply enter the structure (Pb distribution used as marker). As a matter of fact, in the STEM view of the top region of the sample, the Pb nano-agglomerates are vertically stacked into the TiO_2_ pores. A different scenario is encountered in the bottom part of the layer wherein the Pb is atomically distributed (not aggregated) through the TiO_2_ layer even entering into the nano-pores, as further supported by EDX profiling. The coexistence of atomic and clustered Pb is found in the middle part of the layer.

The results indeed indicate that the perovskite enters the TiO_2_ scaffold with a diffusion profile. This implies that, scaling the gig-lox layer thickness to a value of 150–200 nm represents a strategy to get a uniform distribution of the infiltrated perovskite. This reduction of the layer thickness also positively combines with the high absorption coefficient offered by the perovskite materials^18^,^68^ (the dye, instead, having a relatively low absorption coefficient, forces the TiO_2_ thickness to be more properly around 10 μm to increase the density of light absorbers). As a matter of fact, the penetration depth of light in CH_3_NH_3_PbI_3_ layers at 750 nm (close to the bandgap) is ∼250 nm: wavelengths shorter than that value have progressively smaller penetration depth.

**Figure 10:**
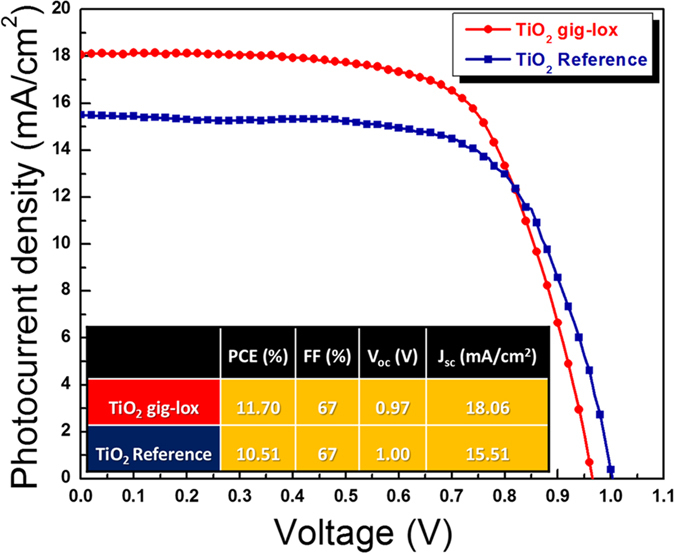
I–V curves of Perovskite Solar Cells. Devices based on gig-lox TiO_2_ layers (∼150 nm-thick) under 1.0 sun illumination (both are reverse curves).

For device purposes, we were indeed authorized to scale the layer thickness to 150 nm. The related cell performances are shown in Fig. 10.

The solar cell based on gig-lox TiO_2_ exhibited a photoconversion efficiency of 11.7%. compared to a device fabricated using commercially available colloidal TiO_2_ grains, which reached 10.5% efficiency. The curves do not show hysteresis.

The enhanced photovoltaic performances of the gig-lox TiO_2_ are mainly ascribed to the higher photocurrent density related to the fine grain morphology of the scaffold. In fact, the gig-lox TiO_2_ layer is characterized by small interconnected grains, as seen in Figs 5 and 9. An additional value-added for the benefit of the device performances is given by the capillary pervasive infiltration of the perovskite into the multi-scale-pores of the gig-lox structure (Fig. 9); this leads to a more extended TiO_2_-perovskite interface that rises the electrons injection efficiency. Although this value does not represent a record^24^, it states the potentialities of the material and encourages further optimizations of the TiO_2_-perovskite blend and of the overall cell architecture.

## Conclusions

We grow multi-porosity TiO_2_ scaffolds offering, in perspective, a transversal empowering over different technologies, including photovoltaics, sensors, water purification, water splitting etc. The method used is new, up-scalable, reliable and indeed industrially implementable, and can be, in principle, extended to any reactive metallic source to produce porous oxides. The multi-scale-porosity (nano and meso) represents the key parameter to allow material of different size (and nature) intimately entering into the TiO_2_ scaffold and forming an active interacting (chemically and physically) blend such as with: dye molecules with their steric hindrance and their anchoring groups; hybrid perovskites with their own chemistry and nanostructuring. The material gains an average porosity of ∼40% in volume which is maintained up to (at least) 500 °C of post-deposition annealing; additionally, surface faceting represents an added-value for the TiO_2_ surface to interact with functional materials. In all those respects, the gig-lox layer structure surpasses what achievable by sputtering in conventional ways. Its competition level with chemically synthesized reference counterparts is doubly demonstrated by integration of the gig-lox TiO_2_ scaffold in DSC and PSC architectures. A high photo-active molecular infiltration was proved by chemisorption of N-719 (∼1 × 10^20^ molecules/cm^3^) on the TiO_2_ surfaces. In Perovskite Solar Cells, the capillary infiltration of CH_3_NH_3_PbI_3_ into the differently sized pores of the TiO_2_ architecture allowed reaching efficiency values of 11.7%, higher than in the reference cell made with commercial TiO_2_ under the same conditions.

## Materials and Methods

### Sputtering equipment

TiO_2_ layers have been deposited on corning glass slices by using two different geometries: a standard parallel plate geometry (Kenotec equipment–Magnetron DC-reactive sputtering) and a customized grazing incidence geometry (Kenosistec equipment–Magnetron DC-pulsed sputtering). The processes on both equipments were calibrated on the basis of the semi-empirical Thornton’s model (T_melting anatase_ = 1843 °C) with the intent of confining the TiO_2_ growth within the porous region (see Fig. 1(a)). Different parameters were thus explored, namely: the anode-cathode distance, the flow rate of both the carrier gas (Ar) and reactive gas (O_2_), the power applied to the cathodes and the temperature on the substrate. The total pressure and the deposition temperature were chosen as shown in Fig. 1(a), and the oxygen partial pressure was tuned in order to get stoichiometric TiO_2_ layers. In both working geometries, each deposition process is preceded by a pre-sputtering step to clean up the surface of the Titanium target and to remove residual thin oxide layer. With our best process, mesoporous TiO_2_ layers were deposited at room temperature, with thickness in the range from 350 to 1000 nm. The effect of post-deposition thermal treatments was also explored, namely at 200 °C for 30 minutes and at 500 °C for 30 (and also 450 °C 1 h) seconds both performed in a mixture of 78%N_2_:22%O_2_.

### TiO_2_ deposition by grazing incidence geometry assisted by local oxidation (gig-lox)

We used a customized grazing incidence source of Titanium (2-inch circular target) at incident angle of 12.7° taken with respect to the sample surface. The choice of the incident angle was done on the basis of geometrical consideration, being a compromise between the deposition rate and the shadowing effectiveness. The other peculiarity of the equipment resides in the oxygen source location, being independent from the Ar source and placed in close proximity to the substrate. A schematic of the equipment is shown in Fig. 1(b). In our optimized process the Ar flow-rate was settled at 69 sccm, corresponding to a deposition pressure of 14.0 μbar (10.5 mTorr-see working region inside the Thornton schematic of Fig. 1(a)). The depositions were done at room temperature in O_2_ reactive ambient with a flow rate of 2 sccm, using a power of 140 W, a current of 475 mA and a voltage of 295 V (power loading 6.9 W/cm^2^). The corresponding growth rate was 4 nm/min, able to guarantee the proper layer stoichiometry (as demonstrated by X-ray photoelectron spectroscopy analyses) by local oxidation during growth. The anode-cathode distance was set at 1.2 cm and, during deposition, the substrate was kept rotating under the beam (20 rpm) in order to improve the layer uniformity over the sample surface. In these working conditions, a double regime is settled up into the deposition chamber (see Fig. 1(b)): a blue plasma (metallic) in proximity of the source, and an oxidizing region confined at the sample surface (violet colour). This allowed avoiding charging effects and working under stable conditions, at relatively high deposition rate. As main focus, the procedure allows performing a progressive local oxidation during growth, thus avoiding *ex-situ* treatments in oxygen. The material used in the paper is the result of systematic action on the deposition parameters (see SI).

### TiO_2_ deposition by parallel plate geometry (ppg)

We used a standard anode-cathode parallel plate equipment with a 6-inch circular target of Titanium as a source. The process was carried out in Oxygen ambient by applying a constant power of 600 W (1.24 A, 498 V, power loading 4.9 W/cm^2^). An O_2_/Ar flow rate ratio as low as of 5/45 sccm was set in order to maintain a growth rate comparable to that used in the gig-lox geometry (here 3.7 nm/min) and to guarantee the proper layer stoichiometry. The deposition processes were carried out at a pressure of 9.0 μbar (7 mTorr-see working region inside the Thornton schematic of Fig. 1a) with an anode–cathode distance of 10 cm. In the chamber, a uniform plasma (pink colour), rich of oxygen species, was established between the plates.

### Spectroscopic Ellipsometry analyses

Spectroscopic Ellipsometry (SE) data were collected using a J.A.Woollam VASE instrument. Measurements were performed in a vertical configuration, which is better suited for transparent samples in order to measure on the same point ellipsometric and transmittance data. Optical spectra were recorded from 300 to 2100 nm (step 5 nm) at 55°, 60° and 65°. An initial model of the optical transitions was built for each layer constituting the sample. The TiO_2_ layer was modeled by using a single Tauc-Lorentz oscillator and the surface roughness by the Bruggeman effective medium approximation (EMA). We evaluated that the use of graded layer model by an increased number of fitting parameters easily leads to unphysical results. In order to fit the sample with absorbed N-719 dye in the spectral range from 2 eV to 3.25 eV, where the transmittance drops abruptly, we have used two additional Gaussian oscillators accounting for the N-719 absorption bands centered at 2.33 eV and 3.15 eV. Special care has been taken to evaluate the optical constants for the glass substrate taking into account backside reflection and unpolarized light. Excellent data fitting has been obtained through the whole range of measurements (300–2100 nm) including transmittance data. From the fitting of the raw psi and delta data (Fig. 4(a,b)) we extracted the optical parameters n and k (the real and imaginary parts of the complex refractive index). The material porosity was measured using the same approach applied by Richards *et al*.^49^, assuming that the nano-structuring (mainly grain size and stoichiometry) of the layer does not remarkably change with the annealing (as verified for the gig-lox layer). This allowed exploring the effect of the thermal treatment on the layer porosity.

### Structural and morphological characterizations

X-ray diffraction (XRD) analyses were performed by using a D8-Discover Bruker AXS diffractometer, equipped with a Cu K_α_ source, in symmetric configuration (source and detector move by the same incremental angle). Transmission Electron Microscopy (TEM) and Selected Area Electron Diffraction analyses (SAED) were done using a JEOL JEM 2010 microscope operating at 200 kV. Field Emission Scanning Electron microscopy (FE-SEM) images were obtained by using a ZEISS VP 55 microscope equipped with Energy Dispersive X-ray analyses (EDX) INCA-Oxford windowless detector. STEM analysis are obtained using a Jeol ARM200 equipped with a cold FEG electron source, CEOS condenser aberration corrector and 100 mm^2^ Jeol EDXS detector. Some images are acquired in STEM mode in Z-contrast configuration. EDXS profiles are extracted by a 730 × 180 nm spectrum image across the whole thickness, with 7 nm pixel size, and 0.1 s pixel time. The scanned area was selected in a thicker region of the sample in order to increase and average the signal.

### Dye-sensitizing, XPS and UV-Vis characterization of the TiO_2_ layers

The surface stoichiometry of the as deposited layers was investigated by X-Ray Photoelectron Spectroscopy (XPS). XPS analyses have been performed by using a PHI ESCA/SAM 5600 Multy technique spectrometer equipped with a Mg K_α_ X-ray source at a pressure of 5 × 10^−9^ Torr. The TiO_2_ layers were sensitized with di-tetrabutylammonium cis-bis (isothiocyanato) bis (2,2-bipyridyl-4,4-dicarboxylato) ruthenium(II) (N-719, Aldrich) by immersion into 5 × 10^−5^ M Ethanol solutions of the complex for 18 h at room temperature. The sensitized layers were withdrawn from the solution, rinsed in pure Ethanol to remove any physisorbed dye and dried in nitrogen flux. UV-VIS measurements were carried out on an UV-Vis V-650 JASCO spectrophotometer, and the spectra were recorded with a ±0.2 nm resolution.

### DSC architecture and I-V characterization

Fluorine-doped tin oxide (FTO, 15 Ω/sq, provided by Solaronix S.A.) glass plates were first cleaned in a detergent solution using an ultrasonic bath for 15 min, and then rinsed with water and ethanol. Compact TiO_2_ layers were prepared as follows: FTO glasses were coated with 0.15 M titanium diisopropoxide bis(acetylacetonate) (75% Aldrich) in 1-butanol (Aldrich) solution by the spin-coating method, which was heated at 125 °C for 5 min. After the coated film was cooled down to the room temperature, the same process was repeated and the two times coated FTO glasses were finally heated at 500 °C for 30 min. The prepared dense TiO_2_ blocking layer were thus covered by: 1) mesoporous TiO_2_ gig-lox layers deposited by sputtering at RT; or 2) standard nanocrystalline TiO_2_ pastes (reference) deposited by a doctor-blading technique using a commercial diluted colloidal titania paste (Dyesol 18NR-T). The reference samples were heated in air at 150 °C for 15 min. To compensate the limitations associated to the poor light harvesting capability^29^ of the sensitizer and to reinforce the potentiality of the new material, we associated to the gig-lox scaffold to a commercial scattering layer by doctor blading a commercial scattering paste (Solaronix D/SP colloidal paste) onto the above described transparent layers and sintering the double-layer photoanode at 500 °C for 30 min. The same was done for the reference photoanode. For all the samples, the dye sensitization was performed by keeping the electrodes for 14 h and under dark in 0.2 mM solutions of bis(tetrabutylammonium)-cis-di(thiocyanato)-N,N′-bis(4-carboxylato-4′-carboxylic acid-2,2′-bipyridine) ruthenium(II) (N-719, purchased by Solaronix S.A.) in a mixture of acetonitrile and tert-butyl alcohol (1:1 v/v). The counter-electrodes were prepared by sputtering a 50 nm Pt layer on a hole-drilled cleaned FTO plate. The photo-anode and the counter-electrode were faced and assembled using a suitably cut 50 μm thick Surlyn^®^ hot-melt gasket for sealing. The iodine redox electrolyte (0.1 M LiI, 0.03 M I_2_, 0.6 M 1-methyl-3-propylimidazolium iodide, and 0.5 M tert-butylpyridine in dry acetonitrile) was vacuum-injected into the space between the electrodes through pre-drilled holes on the back of the counter electrode. The holes were eventually sealed using Surlyn^®^ hot melt film and a cover glass. Photocurrent-voltage measurements were performed using a Keithley unit (Model 2400 Source Meter). A Newport AM 1.5 Solar Simulator (Model 91160 A equipped with a 1000 W Xenon arc lamp) serving as a light source.

### PSC architecture and I-V characterization

Fluorine-doped tin oxide (FTO, 15 Ω/sq, provided by Solaronix S.A.) glass plates were first etched with zinc powder and HCl 2 M to form the desired electrode pattern, then cleaned in a detergent solution using an ultrasonic bath for 15 min, and rinsed with water and ethanol. A hole blocking layer of TiO_2_ (40 nm) was deposited on the cleaned FTO substrates by sputtering.

The TiO_2_ blocking layers were covered by: (1) mesoporous TiO_2_ gig-lox layers deposited by sputtering at RT; or (2) standard nanocrystalline TiO_2_ pastes (reference) deposited by spin coating a commercial diluted colloidal titania paste (Dyesol 30NR-T). The samples were heated in air at 450 °C for 1 h. The annealing is equivalent to 500 °C 30 s.

The perovskite films were deposited from a precursor solution containing 461 mg of PbI_2_, 159 mg of CH_3_NH_3_I, and 78 mg of DMSO (molar ratio 1:1:1) in 600 mg of DMF. The completely dissolved solution was spin-coated on the TiO_2_ layer at 4000 rpm for 25 sec and 100 μL of toluene were poured on the spinning substrate 15 s prior to the end of the program. The film were heated at 65 °C for 1 min and 100 °C for 2 min in order to obtain a dense CH_3_NH_3_PbI_3_ film^69^. The hole transporting material solution, Spiro-MeOTAD, 50 mM in chlorobenzene containing 25 mM bis(trifluoromethylsulfonyl)-imide lithium salt (Li-TFSI) and 200 mM 4tert-butylpyridine (TBP) was spun at 3000 rpm for 30 s. As a last step, 60 nm of gold top electrode were thermally evaporated under high vacuum. Photocurrent-voltage measurements were performed using a Keithley unit (Model 2400 Source Meter). A Newport AM 1.5 Solar Simulator (Model 91160A equipped with a 1000W Xenon arc lamp) serving as a light source.

## Additional Information

**How to cite this article**: Sanzaro, S. *et al*. Multi-Scale-Porosity TiO_2_ scaffolds grown by innovative sputtering methods for high throughput hybrid photovoltaics. *Sci. Rep.* **6**, 39509; doi: 10.1038/srep39509 (2016).

**Publisher's note:** Springer Nature remains neutral with regard to jurisdictional claims in published maps and institutional affiliations.

## Supplementary Material

Supplementary Information

## Figures and Tables

**Table 1:** Porosity calculated by Ellipsometric measurements.

	Parallel plate geometry		Grazing incidence geometry assisted by local oxidation		
	As deposited	Annealed at 500 °C	As deposited	Annealed at 500 °C	Annealed at 500 °C + N-719
Refractive index at 550 nm	2.22	2.30	2.13	2.05	2.22
Porosity (%)	34	≤21	41	41	28

Refractive index and layer porosity from Ellipsometric data fitting.
