# Supplementary material for: Multi-Scale-Porosity TiO2 scaffolds grown by innovative sputtering methods for high throughput hybrid photovoltaics
Source: Sci Rep. 2016 Dec 21;6:39509. doi: 10.1038/srep39509 (PMC5175132; doi:10.1038/srep39509)
Supplement: Supplementary Information [file srep39509-s1.pdf]

Scientific Report

Supplementary Information

Multi-Scale-Porosity TiO_2 scaffolds grown by innovative sputtering methods for high throughput hybrid photovoltaics

Salvatore Sanzaro[1,2], Emanuele Smecca[1], Giovanni Mannino[1], Corrado Bongiorno[1], Giovanna Pellegrino[1], Fortunato Neri[2], Graziella Malandrino[3], Maria Rita Catalano[3], Guglielmo Guido Condorelli[3], Rosabianca Iacobellis[4,5], Luisa De Marco[6], Corrado Spinella[1], Antonino La Magna[1] and A. Alberti[1,*]

[1] National Research Council-Institute for Microelectronics and Microsystems (CNR-IMM), Zona Industriale - Strada VIII n°5, Catania 95121, Italy.
[2] Department of Mathematical and Computational Sciences, Physics and Earth Sciences, University of Messina, V. le F. Stagno d'Alcontres 31, Messina 98166, Italy.
[3] Department of Chemical Sciences, University of Catania, V. le Andrea Doria 6, 95125 Catania, Italy.
[4] Center for Biomolecular Nanotechnology (CBN) Italian Institute of Technology Foundation, Via Barsanti sn, 73010, Arnesano, Italy.
[5] Department of Innovation Engineering, University of Salento, Via per Monteroni 73100, Lecce, Italy.
[6] Institute of Nanotechnology National Research Council (CNR-Nanotec), District of Technology, Via Arnesano 16, 73100 Lecce, Italy.

*alessandra.alberti@imm.cnr.it

Additional information on the gig-lox process

Our approach exploits: 1) a high deposition rate (4nm/min) due to the metallic plasma established at the source side (charging effects by surface oxidation are indeed avoided); 2) the progressive local oxidation of the landing species at the anode side; 3) the shadowing effect by the starting seeds due to the inclined (off-axis) Ti fluxes by a θ angle. These expedients provide the material with an additional meso-porosity (besides the nano-porosity arising from the Thornton's model) that is maintained during (eventually needed) post-deposition thermal treatments (see hereafter for a detailed description). As a consequence of the species separation (titanium and oxygen), the plasma gains a double color as shown in in Fig. 1(b) (blue for Ti-rich plasma, violet for Oxygen-containing plasma). The optimization on the θ angle is shown in figure 1S.

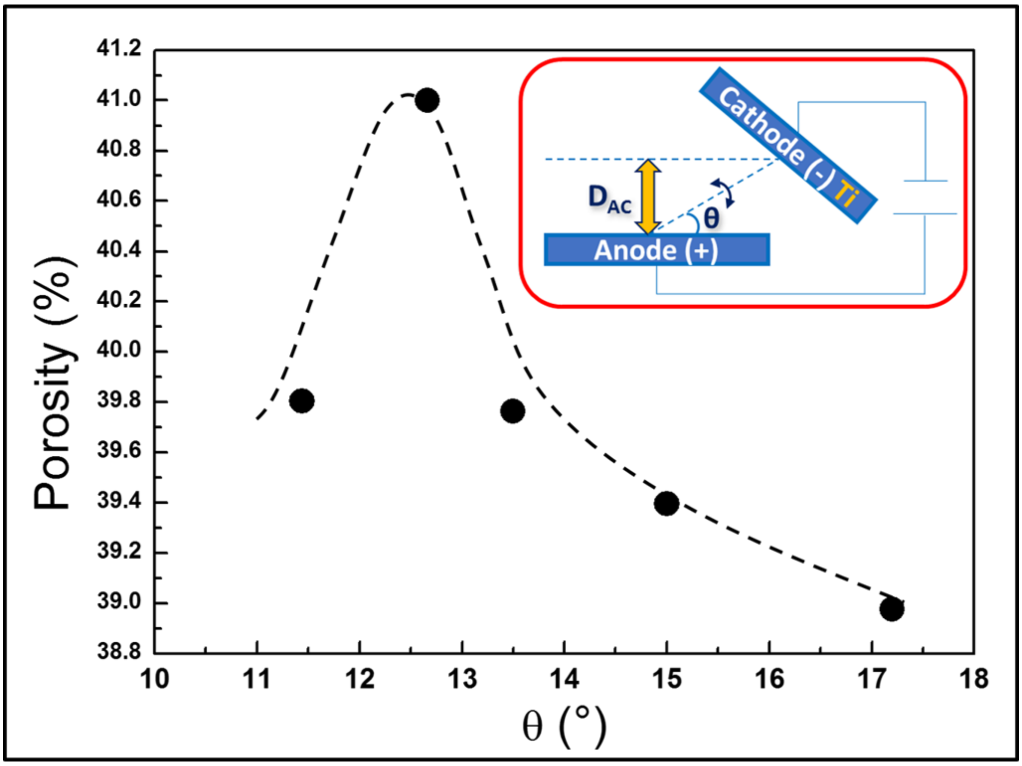

Figure 1S. – TiO_2 layer porosity as a function of the inclination angle (θ) of the Ti source with respect to the sample position. Data collected at 20 rpm and on the as deposited samples; the layer thickness is ~800nm.

Grain population and diameter distribution

The material used in the paper is the result of systematic action on the deposition parameters. Among the others, hereafter we report the effect of changing the rotational speed on porosity and grain size (the parameters of interest). The grain population and diameter distribution are shown in Figure 2S. Working in static condition was a priori excluded due to a lack of thickness uniformity over the sample surface. On the basis of our combined results, the best compromise between small grain size and high porosity was considered that one used in the paper, corresponding to 20 rpm of rotational speed. At this rotation speed, the effect of the inclination angle was evaluated, as discussed in the paper.

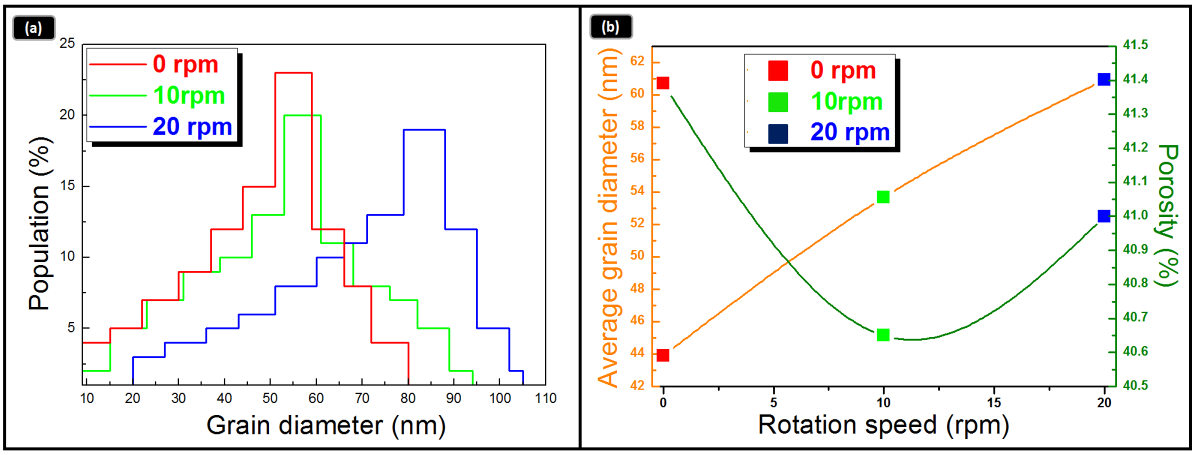

Figure 2S. – a) TiO_2 grain diameter distribution; b) average grain diameter and layer porosity of the TiO_2 layer as a function of the rotation speed.

XPS analyses. The binding energy (BE) scale was calibrated by centering the C 1s signal, due to the adventitious/hydrocarbon carbon, at 285.0 eV. The XPS wide spectrum performed on the N-719 functionalized TiO_2 layers (3Sa) shows the presence of the expected elements: nitrogen, carbon, ruthenium, sulfur, oxygen and titanium. The titanium contribution (figure 3Sb) shows the typical spin-orbit doublet with the Ti $2p_{3/2}$ component centered at 458.2 eV and Ti $2p_{1/2}$ component centered at 464.3 eV. The O 1s signal (3Sc) shows a main component at 530.2 eV attributed to TiO_2 and a shoulder at 532.1 eV due to Ti–OH groups[1]. The Ti:O atomic ratio is ~1:2.2. The not perfect accordance with the oxide stoichiometric value is likely related to the presence of the hydroxyl groups on the surface. The result is independent of the deposition method.

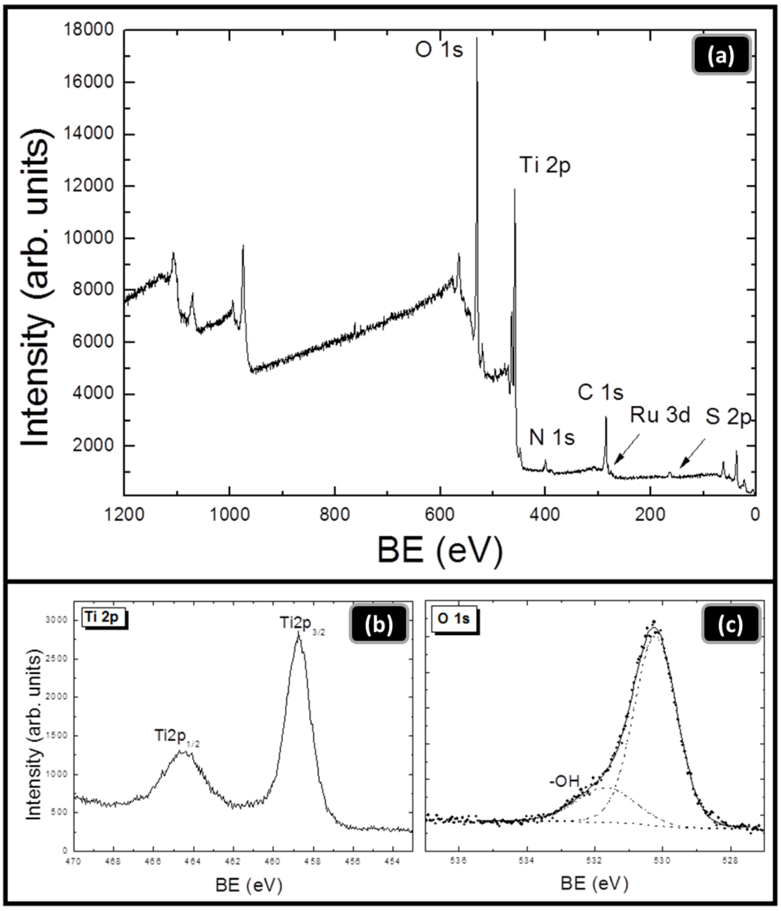

Figure 3S. XPS analysis: a) wide scan spectra b) Titanium and c) Oxygen contributions to determine the titanium oxide stoichiometry in as deposited condition, bringing out as the Ti:O atomic ratio is not perfect due to the presence of some OH groups on the TiO_2 surface.

Absorption coefficient

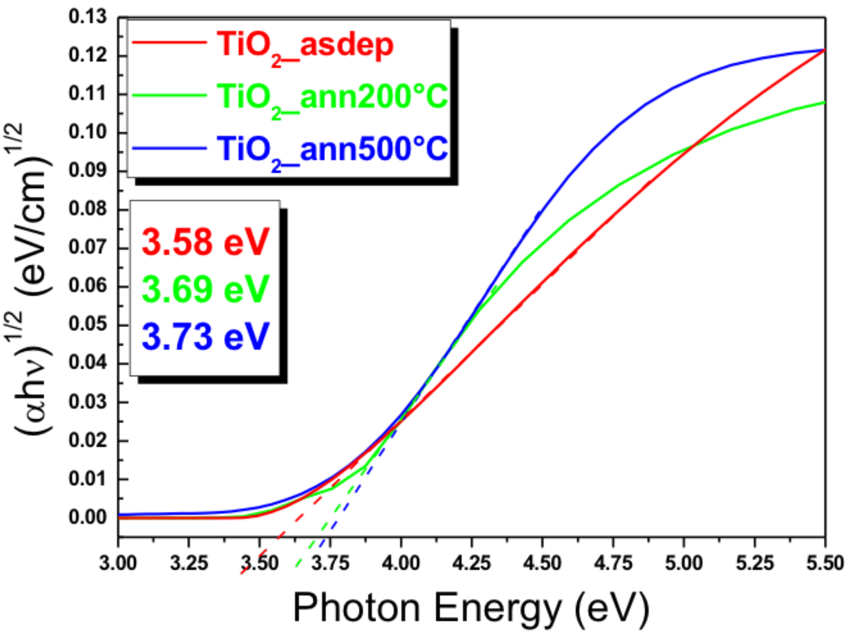

Figure 4S. – Tauc plots of the absorption coefficient elucidating the effect of the annealing on of the TiO_2 gig-lox layer in improving the lattice structure.

XRD analysis. Fig. 5S shows the progressive nanostructuring of the TiO_2 gig-lox layer in the anatase form. This structural improvement has effect on the optical bandgap but is ineffective at the side of the layer porosity. The Full Width at Half Maximum (FWHM) of the diffraction peaks was used to calculate the size of the anatase domains. In particular, in the gig-lox layer FWHM values of 0.41° and 0.49° were measured for (101) and (004) reflections, respectively; in the ppg layer, instead, the FWHM for the same set of peaks is 0.52° and 0.44°. The domain size was thus calculated by the Scherrer's formula[2], being in average ~18±1 nm for the gig-lox layer and ~17±1 nm for ppg layer (see Table 1S). Both values settle around the size expected for the anatase that is more stable in nanostructures with respect to the rutile counterpart. Please remember that the grain size evaluated by X-ray diffraction accounts for small un-defective domains coherently diffracting the incident beam. They do not necessarily match the grains as defined by morphological analyses (e.g. by FE-SEM).

Table 1S: XRD data for the TiO_2 anatase peaks in both deposition geometries.

TiO_2 XRD Analysis							
		Grazing Incidence Geometry assisted by Local Oxidation			Parallel Plate Geometry		
	hkl	2θ (deg)	FWHM (deg)	Grain Size (nm)	2θ (deg)	FWHM (deg)	Grain Size (nm)
Simmetric diffraction - (2θ-ω)	(101)	25,38	0,41	19,9	25,28	0,52	15,7
Simmetric diffraction - (2θ-ω)	(004)	37,94	0,49	17,2	37,88	0,44	19,1
REFERENCE		$2\theta_{(101)}$ = 25,28°<br>$2\theta_{(004)}$ = 37,80°					

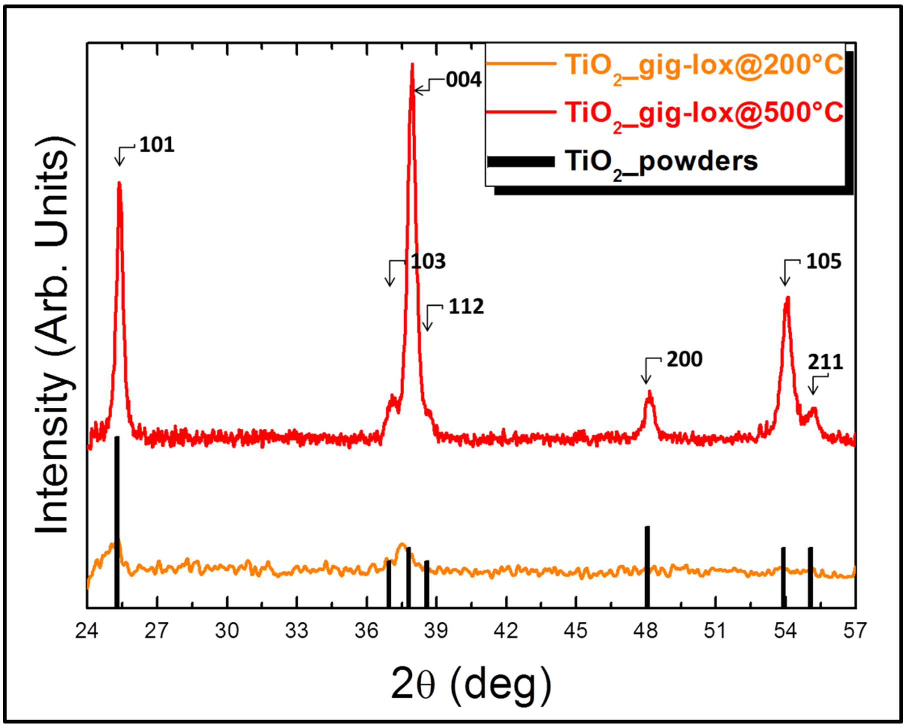

Figure 5S. Diffraction patterns of the gig-lox layer after annealing at 200°C and 500°C. With respect to the as deposited material, the nano-grains have gained a partial degree of order at 200°C with their consequent cooperative participation to some diffraction peaks. A full rearrangement of the mesocrystals in anatase is gained at 500°C.

Table 2S: Texturing coefficient for TiO_2 anatase peaks in both deposition geometries. The texturing coefficients is calculated on the basis of the formula[3] $J = \frac{I_{hkl}}{I^*_{hkl}} / \sum_{hkl}\left(I_{hkl}/I^*_{hkl}\right)$ (where, I^*_{hkl} is the expected intensity in a reference powder.

TiO_2 Texturing Coefficient							
Planes	I*	grazing incidence geometry assisted by local oxidation			parallel plate geometry		
		I	I/I*	J	I	I/I*	J
(101)	100,00	976,20	9,76	0,066	975,20	9,75	0,083
(103)	10,00	166,50	16,65	0,113	215,80	21,58	0,183
(004)	20,00	1420,60	71,03	0,482	825,40	41,27	0,350
(112)	10,00	108,80	10,88	0,074	100,60	10,06	0,085
(200)	35,00	192,00	5,49	0,037	219,20	6,26	0,053
(105)	20,00	544,40	27,22	0,185	427,60	21,38	0,181
(211)	20,00	124,70	6,24	0,042	152,80	7,64	0,065
		$\Sigma(I/I^*)$ =	147,26		$\Sigma(I/I^*)$ =	117,94	

UV-Vis optical absorption analyses. Table S3 shows the net absorbance and the related density of N-719 molecules infiltrated inside the mesoporous materials as a function of the annealing temperature.

Table 3S: Molecular density evaluated by UV-Vis measurements and estimated by the Lambert-Beer's law.

TiO_2 UV-Vis Measurements				
**Deposition Geometry**	**Temperature (°C)**	**Thickness (nm)**	**Net Absorbance**	**Molecules/cm^3**
Grazing Incidence + Local Oxidation	200	800±10	0,098	$7,38*10^{19}$
	500		0,128	$9,63*10^{19}$
Parallel Plate	200	500±15	0,027	$3,25*10^{19}$
	500		~0	~0

REFERENCES

1. Pellegrino, G., et al. A strategy to stabilise the local structure of Ti^{4+} and Zn^{2+} species against aging in TiO_2/aluminium-doped ZnO bi-layers for applications in hybrid solar cells. *Journal of Applied Physics* **116**, 054907 (2014).

2. Cullity, B.D. *Elements of X- ray diffraction*, 2nd Edition Addison Wesley, Reading, MA, USA **1978**.
